# The active site of hen egg-white lysozyme: flexibility and chemical bonding

**DOI:** 10.1107/S1399004714001928

**Published:** 2014-03-21

**Authors:** Jeanette Held, Sander van Smaalen

**Affiliations:** a Laboratory of Crystallography, University of Bayreuth, D-95440 Bayreuth, Germany

**Keywords:** hen egg-white lysozyme, multipole model, multipole parameters

## Abstract

Chemical bonding at the active site of hen egg-white lysozyme (HEWL) is analyzed on the basis of Bader’s quantum theory of atoms in molecules [QTAIM; Bader (1994 ▶), *Atoms in Molecules: A Quantum Theory.* Oxford University Press] applied to electron-density maps derived from a multipole model. The observation is made that the atomic displacement parameters (ADPs) of HEWL at a temperature of 100 K are larger than ADPs in crystals of small biological molecules at 298 K. This feature shows that the ADPs in the cold crystals of HEWL reflect frozen-in disorder rather than thermal vibrations of the atoms. Directly generalizing the results of multipole studies on small-molecule crystals, the important consequence for electron-density analysis of protein crystals is that multipole parameters cannot be independently varied in a meaningful way in structure refinements. Instead, a multipole model for HEWL has been developed by refinement of atomic coordinates and ADPs against the X-ray diffraction data of Wang and coworkers [Wang *et al.* (2007), *Acta Cryst.* D**63**, 1254–1268], while multipole parameters were fixed to the values for transferable multipole parameters from the ELMAM2 database [Domagala *et al.* (2012), *Acta Cryst.* A**68**, 337–351] . Static and dynamic electron densities based on this multipole model are presented. Analysis of their topological properties according to the QTAIM shows that the covalent bonds possess similar properties to the covalent bonds of small molecules. Hydrogen bonds of intermediate strength are identified for the Glu35 and Asp52 residues, which are considered to be essential parts of the active site of HEWL. Furthermore, a series of weak C—H⋯O hydrogen bonds are identified by means of the existence of bond critical points (BCPs) in the multipole electron density. It is proposed that these weak interactions might be important for defining the tertiary structure and activity of HEWL. The deprotonated state of Glu35 prevents a distinction between the Phillips and Koshland mechanisms.

## Abstract 2

Chemical bonding at the active site of lysozyme is analyzed on the basis of a multipole model employing transferable multipole parameters from a database. Large *B* factors at low temperatures reflect frozen-in disorder, but therefore prevent a meaningful free refinement of multipole parameters.

## 1. Introduction   

**Figure 1:**
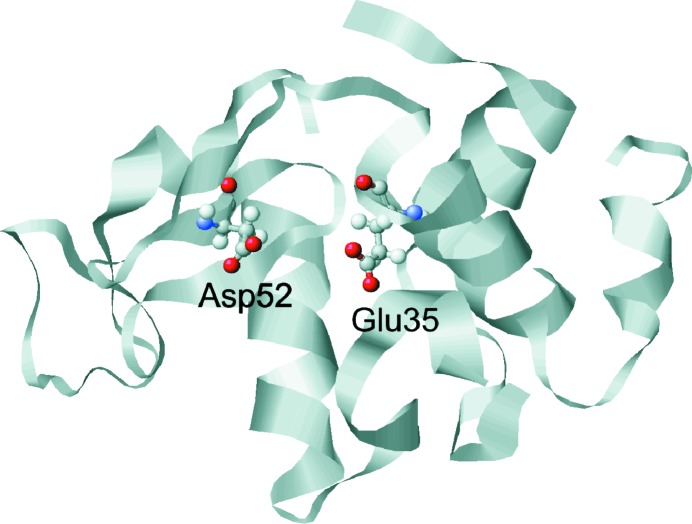
Schematic drawing of Glu35 and Asp52 within HEWL.

The three-dimensional structure of the antibacterial hen egg-white lysozyme (HEWL) was uncovered in 1965 (Blake *et al.*, 1965 ▶), heralding enzyme crystallography. Today, lysozyme resides among the most intensively studied enzymes and its function is well known. Lysozyme damages bacterial cells by cleaving the β(1→4) glycosidic linkage between alternating units of *N*-acetylmuramic acid and *N*-acetylglucosamine, which are the building blocks of bacterial cell walls. According to the Phillips mechanism (Phillips, 1966 ▶), the two residues Glu35 (glutamic acid) and Asp52 (aspartic acid) (Fig. 1 ▶) play an essential role. The terminal proton of Glu35 is transferred to the O atom of the glycosidic bond between two neighbouring sugar residues, leading to the cleavage of the glycosidic bond and the formation of a carbenium ion. The positive charge of this carbenium ion is stabilized by the negative charge of Asp52 until a hydroxyl ion binds to the positive C atom and Glu35 is reprotonated.

Another reaction mechanism, proposed by Koshland (1953 ▶), involves a glycosyl-enzyme intermediate in which the substrate is covalently bonded to Asp52. Vocadlo *et al.* (2001 ▶) concluded in their electrospray-ionization mass-spectrometric study that the catalytic mechanism of lysozyme involves an intermediate complex in which one sugar ring is covalently bonded to Asp52. A crystallographic study at 1.5 Å resolution of HEWL crystallized with substrate by Strynadka & James (1991 ▶) supports the Phillips mechanism (Phillips, 1966 ▶) and does not indicate a covalent intermediate. They pointed out the environment of Glu35 and Asp52, which among other things is constructed by hydrogen bonds that can assist the catalysis according to Phillips (1966 ▶).

An important factor for the catalytic or other functions of a protein is its flexibility (Branden & Tooze, 1999 ▶). All atoms are moving in a temperature-dependent breathing motion, which is retained in crystals. In crystal structures, flexible parts appear as disorder or give rise to large values of *B* factors, while these regions attain low levels of electron density or almost no density at all (Branden & Tooze, 1999 ▶). Analysis of crystallographic *B* factors thus allows inferences concerning the relations between structure and dynamics of proteins (Frauenfelder *et al.*, 1979 ▶; Artymiuk *et al.*, 1979 ▶; Karplus & Schulz, 1985 ▶; Carugo & Argos, 1999 ▶; Parthasarathy & Murthy, 2000 ▶; Stocker *et al.*, 2000 ▶; Radivojac *et al.*, 2004 ▶; Yuan *et al.*, 2005 ▶; Weiss, 2007 ▶).

Apart from flexibility, the stability and the function of proteins primarily depends on chemical interactions. The electron density in the unit cell can provide qualitative and quantitative information on chemical interactions of molecules, including proteins (Gatti & Macchi, 2012 ▶). This encompasses information on the degree of covalency of bonds, on the nature of hydrogen bonds and on covalent and electrostatic interactions, opening the door for a detailed insight into protein structure, function and potentially to drug design.

Electron-density studies are an established tool for extracting information about chemical interactions involving small molecules, and thus can shed light on the functionality regarding chemical stability, chemical reactions and physical properties. Electron-density studies are usually based on refinement of the parameters of the multipole model against X-ray diffraction data (Hansen & Coppens, 1978 ▶). The multipole model is an extension of the independent atom model (IAM) by many functions and many parameters that describe the rearrangements of electrons as occur, for example, upon the formation of chemical bonds (Coppens, 1997 ▶). Refinements of multipole parameters are only possible if accurate X-ray diffraction data have been measured at low temperature (*T* < 100 K) and to high resolution (*d*_min_, preferably below 0.50 Å). A single protein (crambin; Jelsch *et al.*, 2000 ▶; Schmidt *et al.*, 2011 ▶) diffracts to this limit, while subatomic resolution data sets of several more proteins have been measured (Ko *et al.*, 2003 ▶; Howard *et al.*, 2004 ▶; Wang *et al.*, 2007 ▶). Here, we present an analysis of the *d*_min_ = 0.65 Å data set of HEWL as published by Wang *et al.* (2007 ▶).

The current and developing state of the art of X-ray diffraction promises more high-resolution, high-quality data of proteins in the future (Duke & Johnson, 2010 ▶; Dauter *et al.*, 2010 ▶; Mueller *et al.*, 2012 ▶; Garman & Weik, 2013 ▶). One factor limiting the number of very high-resolution data sets in the Protein Data Bank (PDB; Berman *et al.*, 2000 ▶) is the geometry of many diffractometers for protein diffraction. With an area detector placed perpendicular to the beam, and a maximum angle of incidence onto the detector of 60° for good-quality data, the resolution is given by the wavelength of the radiation, *d*_min_ = λ, and is usually worse than 0.50 Å, even if the crystal could diffract better. Therefore, diffractometers with an area detector that can be rotated to offset positions in 2θ are highly desirable to obtain high-quality diffraction data at subatomic resolutions.

Information on chemical interactions is contained in the topological properties of static electron densities, where the latter are electron densities after the removal of all thermal motion. They are obtained from a structure model with all atomic displacement parameters (ADPs) set to zero, resulting in the deconvolution of electron density and atomic displacements. Topological properties of static electron densities are usually interpreted according to the quantum theory of atoms in molecules (QTAIM; Bader, 1994 ▶). This method has been extensively applied to crystals of amino acids and small peptides, especially tripeptides (Jelsch *et al.*, 1998 ▶; Destro *et al.*, 2000 ▶; Flaig *et al.*, 2002 ▶; Rödel, 2003 ▶; Scheins *et al.*, 2004 ▶; Dittrich *et al.*, 2005 ▶; Mebs *et al.*, 2006 ▶; Checińska *et al.*, 2006 ▶; Kalinowski *et al.*, 2007 ▶; Johnas *et al.*, 2009 ▶). As opposed to static electron densities, dynamic electron densities employ both the atomic coordinates and the atomic displacements resulting from a structure refinement against diffraction data. Thus, dynamic densities describe a smeared electron-density distribution. Here, we consider dynamic model densities, which are computed by inverse Fourier transformation of a complete set of structure-factor amplitudes and phases that have been calculated from a structure model up to a resolution far beyond experimental access (Mondal *et al.*, 2012 ▶). As a consequence, dynamic model densities are not subject to series-termination effects of the Fourier transform, thus allowing an accurate description of its fine topological properties. The dynamic model densities can be accurately computed for any structure model, including IAM and multipole models. They are essentially different from the dynamic densities sometimes defined as the inverse Fourier transform of the phased experimental structure factors. Although the latter are closer to the experiment, the limited resolution of the experimental data necessarily leads to series-termination effects in the corresponding electron density, thus preventing a meaningful analysis of topological properties.

An increasing size of the molecule is accompanied by an increasing number of parameters to be refined. The assignment of multipole parameters to a protein structure boosts the number of parameters even further, which may limit the refinement of the multipole model. However, it has been found that the multipole parameters of an atom in a particular environment (*e.g.* the C^α^ atom in alanine) are nearly the same in all compounds containing this atom (Pichon-Pesme *et al.*, 1995 ▶). Based on this principle, several databases of transferable multipole parameters have been developed (Pichon-Pesme *et al.*, 1995 ▶, 2004 ▶; Volkov *et al.*, 2004 ▶; Dittrich *et al.*, 2006 ▶; Zarychta *et al.*, 2007 ▶; Dominiak *et al.*, 2007 ▶, 2009 ▶; Domagała *et al.*, 2012 ▶). They offer the possibility of constructing a multipole model of any molecule without the need to refine parameters beyond those of the IAM, *i.e.* only atomic coordinates, ADPs and site occupancies are refined. Electron densities based on multipole models of proteins have previously been considered for crambin (Jelsch *et al.*, 2000 ▶; Schmidt *et al.*, 2011 ▶), human aldose reductase (Guillot *et al.*, 2008 ▶) and trypsin (Schmidt *et al.*, 2003 ▶). Multipole parameters have been refined in these cases, whereas for scorpion toxin (Housset *et al.*, 2000 ▶) a multipole model was developed with multipole parameters fixed to database values. A similar approach was used for a series of neuraminidase–inhibitor complexes (Dominiak *et al.*, 2009 ▶).

The original aim of the present study was to elucidate the reaction mechanism catalyzed by HEWL from consideration of electron-density distributions. We have not achieved this goal yet, mainly because high-resolution X-ray diffraction data are only available for native HEWL (Wang *et al.*, 2007 ▶) and not for protein–substrate complexes.

However, in the course of this study we have obtained several results that we consider important for future electron-density studies of HEWL and of proteins in general, and potentially of other large biomolecules such as DNA or RNA. We present here a multipole model for HEWL. We make the observation that the ADPs of atoms in the ordered part of HEWL have values comparable to or larger than those of serine at 298 K, despite the fact that the diffraction data were measured at *T* = 100 K. We explain this feature as frozen-in disorder, but the implication is that ADPs are too large for a meaningful refinement of the multipole parameters (Dittrich *et al.*, 2009 ▶; Gatti & Macchi, 2012 ▶). Apparently, this feature has been overlooked in previous electron-density studies on proteins (Jelsch *et al.*, 2000 ▶; Schmidt *et al.*, 2003 ▶, 2011 ▶; Guillot *et al.*, 2008 ▶).

Secondly, we provide a characterization of chemical bonding near the active site of HEWL through topological analysis of the static electron density. Following two earlier reports on amino acids (Mondal *et al.*, 2012 ▶; Prathapa *et al.*, 2013 ▶), we present here the dynamic electron density of the IAM and multipole model of HEWL, along with an analysis of their topological properties. Thus, we demonstrate that we can compute dynamic densities of structure models of proteins which are free of series-termination effects.

## 2. Computational details   

X-ray diffraction data of HEWL without substrate, as deposited in the PDB (PDB entry 2vb1; Wang *et al.*, 2007 ▶; Table 1 ▶), were employed for structure refinements of the IAM and of a database-based multipole model. Prior to the structure refinements, a solvent correction of the structure factors according to Phillips (1980 ▶) was performed (Jiang & Brünger, 1994 ▶). Structure refinements were performed with the computer program *MoPro* (Guillot *et al.*, 2001 ▶; Jelsch *et al.*, 2005 ▶) against reflections of the single-crystal X-ray diffraction data, employing the strategy described for human aldose reductase by Guillot *et al.* (2008 ▶).

The structure model of HEWL published by Wang *et al.* (2007 ▶) (PDB entry 2vb1) was employed as the starting model for the IAM refinement. The overall scale factor, the coordinates of ordered non-H atoms with a temperature factor *B* of <9 Å^2^ and the anisotropic ADPs of these atoms were refined alternatingly, thus following the procedure proposed by Guillot *et al.* (2008 ▶). Atoms not included in this subsystem were not varied within the structure refinement. A refinement of all atoms with *B* < 8 Å^2^ led to marginally worse agreement indices, whereas a subsystem of atoms with *B* > 9 Å^2^ would contain heavily distributed atoms that are not suitable for refinement (Guillot *et al.*, 2008 ▶).

H atoms were geometrically constrained and fixed at bond lengths known from X-ray diffraction (Allen, 1986 ▶). The ADPs of H atoms were constrained to values of 1.2 (for C—H and N—H) or 1.5 (for O—H, –CH_3_ and –NH_3_) times the values of their neighbouring atoms (Guillot *et al.*, 2001 ▶; Jelsch *et al.*, 2005 ▶; Müller *et al.*, 2006 ▶).

As the final stage of their refinement, Wang *et al.* (2007 ▶) reported an optimization of the weighting scheme. We have not succeeded in obtaining details of this weighting scheme. Therefore, we omitted the 293 worst-fitting reflections with |*I*_obs_ − *I*_calc_|/σ(*I*_obs_) > 45. A refinement without the employment of this criterion resulted in *R*_F_ = 8.60% for all reflections. The choice of the criterion |*I*_obs_ − *I*_calc_|/σ(*I*_obs_) > 45 reflects the possibility that a few reflections might have obtained inaccurate intensities in the experiment, while by this choice the value of *R*_F_ is lowered to 8.39% (*R*_F_ = 8.54% for all reflections).

Subsequently, C—H, N—H and O—H bonds were elongated towards the bond-length values known from neutron diffraction (Allen & Bruno, 2010 ▶). This procedure is necessary to counteract the known shortening of bond lengths for attached H atoms arising from the shift of the one-electron entity, as observable by X-ray diffraction, away from the true position of the hydrogen nucleus. More accurate atomic positions for H atoms are obtained in this way (Steiner, 2002 ▶). Refinement of the scale factor resulted in the final IAM. At convergence *R*_F_ = 8.39% (*R*_F_ = 8.54% for all reflections), which is equal to the value obtained with X-ray distances.

From this model, the static electron density ρ^stat^_IAM_(**r**) was computed by *VMoPro* from the *MoPro* package. This software generates the electron density as a superposition of atomic electron densities described by the same atomic wavefunctions as employed for computation of atomic scattering factors in the refinement (Guillot *et al.*, 2001 ▶; Jelsch *et al.*, 2005 ▶).

The dynamic IAM density ρ^dyn^_IAM_(**r**) was constructed by superposition of thermally smeared spherical atomic electron densities (as defined by the ADPs) by the computer program *PRIOR* (van Smaalen *et al.*, 2003 ▶; Mondal *et al.*, 2012 ▶). The dynamic density of a structure model is basically a Fourier map of the structure factors, albeit free from the series-termination effects that are necessarily present in bonding regions when computing Fourier maps at any experimental resolution. Actually, the computer program *PRIOR* computes dynamic electron densities by Fourier inversion of a complete set of structure factors up to a resolution of approximately *d*_min_ = 0.08 Å. The structure factors are computed from the structure model (coordinates, ADPs, site occupancy factors and multipole parameters) employing the same atomic wavefunctions as are used in computer programs for multipole refinements (Mondal *et al.*, 2012 ▶).

A multipole model was obtained by transfer of multipole parameters from the ELMAM2 database (Domagała *et al.*, 2012 ▶) to all protein atoms of the final IAM. Refinements were performed alternatingly of the overall scale factor, the atomic coordinates of ordered non-H atoms with a temperature factor *B* < 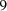
 Å^2^ and the ADPs of these atoms, while all multipole parameters were kept fixed at their database values. The utilization of multipole parameters from the ELMAM2 database leads to a slightly better fit to the data than the fit obtained with the final IAM (Jelsch *et al.*, 1998 ▶; Dittrich *et al.*, 2005 ▶; Kalinowski *et al.*, 2007 ▶; Johnas *et al.*, 2009 ▶; Table 1 ▶). The resulting multipole model is referred to as the ELMAM2 model. By superposition of the aspherical atomic electron densities based on this model, the static electron density ρ^stat^_MP_(**r**) was generated by *VMoPro* in a analogous manner to the computation of ρ^stat^_IAM_(**r**). The computer program *PRIOR* was used to construct the dynamic multipole density ρ^dyn^_MP_(**r**) by superposition of thermally smeared aspherical atomic electron densities in an analogous manner as was performed for ρ^dyn^_IAM_(**r**).

Topological properties of the dynamic electron densities were calculated with the computer program *EDMA* (Palatinus *et al.*, 2012 ▶), while *VMoPro* was employed for topological analysis of the static densities. Topological properties include positions of local maxima of the densities, which are identified with positions of atoms, and atomic basins, which are the volumes around the maxima that are assigned to each atom. The integrated density within a basin provides the atomic charge. Furthermore, the density has saddle points between pairs of atomic maxima, which are called bond critical points (BCPs). According to QTAIM (Bader, 1994 ▶), the existence of a BCP between two maxima indicates a chemical interaction between this pair of atoms. The associated density values ρ(BCP), as well as the values of the second derivatives of the density, as provided through eigenvalues of the Hessian matrix and Laplacians ∇^2^ρ(BCP), can be used to obtain information about the character of the interaction. Kinetic energy densities *G*(BCP) and potential energy densities *V*(BCP) as well as total energy densities *H*(BCP) and the ratio |*V*(BCP)|/*G*(BCP) have been calculated according to the approximate formulas of Abramov (1997 ▶), which allow these quantities to be obtained from ρ(BCP) and ∇^2^ρ(BCP).

## 3. Results and discussion   

### 3.1. Global properties of density maps   

All four electron-density maps are positive everywhere, with the exception of ρ^stat^_MP_(**r**), which contains a few small regions, far away from atoms and bonds, of negative density, with a very small minimum value of −5.4 × 10^−5^ e Å^−3^. Since negative electron densities are nonphysical, their presence in ρ^stat^_MP_(**r**) indicates a shortcoming of the model, which may be the result of using values of the multipole parameters from the ELMAM2 database instead of using their true values. Alternatively, negative densities may indicate a non-perfect deconvolution of static structure and thermal motion owing to the limited resolution of the X-ray diffraction data (Volkov *et al.*, 2007 ▶).

While all local maxima of ρ^dyn^_IAM_(**r**) can be identified with atomic positions, ρ^dyn^_MP_(**r**) contains 38 spurious local maxima in low-density regions. *VMoPro* does not provide this kind of information for static electron densities. We again attribute their occurrence to incompatibilities between neighbouring aspherical atoms from the ELMAM2 database. They do not constitute a problem and have not been considered in the analysis of bonding features, because they occur far outside atoms and bonds (with a distance larger than 3 Å). These spurious maxima possess a total integrated volume of corresponding basins of less than 1% of the volume of the unit cell, and contain an integrated number of electrons smaller than 0.1% of the total number of electrons.

**Figure 2:**
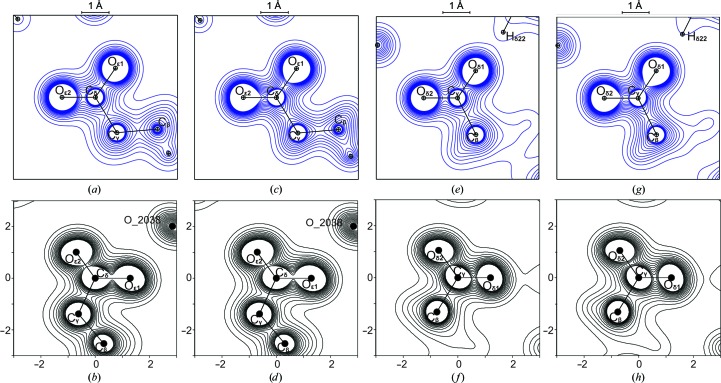
Sections of 6 × 6 Å^2^ through the planes of the carboxyl groups of Glu35 and Asp52. (*a*) ρ^stat^_IAM_(**r**) of Glu35; (*b*) ρ^dyn^_IAM_(**r**) of Glu35; (*c*) ρ^stat^_MP_(**r**) of Glu35; (*d*) ρ^dyn^_MP_(**r**) of Glu35. (*e*) ρ^stat^_IAM_(**r**) of Asp52; (*f*) ρ^dyn^_IAM_(**r**) of Asp52; (*g*) ρ^stat^_MP_(**r**) of Asp52; (*h*) ρ^dyn^_MP_(**r**) of Asp52. Contour lines of equal density are given from 0.2 to 3.0 e Å^−3^ in steps of 0.2 e Å^−3^.

Within the low-density regions only small differences exist between ρ^stat^_MP_(**r**) and ρ^stat^_IAM_(**r**), as shown for Glu35 and Asp52 in Figs. 2 ▶(*a*), 2 ▶(*c*), 2 ▶(*e*) and 2 ▶(*g*). While the static IAM density exhibits nearly spherical contours for ρ(**r**) > 3 e Å^−3^, the static multipole density shows pear-shaped distortions of these contours pointing into the covalent bonds, thus reflecting the description of electron densities within covalent bonds by the ELMAM2 model.

The dynamic electron densities exhibit elliptically distorted densities about the atoms, which are especially apparent for the O atoms of the carboxyl groups (Figs. 2 ▶ *b*, 2 ▶ *d*, 2 ▶ *f* and 2 ▶ *h*). This distinctive feature can be attributed to the anisotropy of the atomic displacements. As opposed to small molecules at *T* < 100 K (Mondal *et al.*, 2012 ▶), differences between IAM and ELMAM2 models are almost obliterated in their dynamic electron densities owing to the large values of the ADPs (§[Sec sec3.2]3.2).

BCPs of covalent non-hydrogen bonds are found at corresponding positions in all four electron-density maps studied in the present work (Tables 2 ▶ and 3 ▶). As explained elsewhere, thermal motion dramatically diminishes the peak values of local maxima, such that some H atoms do not constitute a local maximum in dynamic electron densities, and their electron density is incorporated into the atomic basin of the *X* (C, N or O) atom to which they are bonded (Hofmann *et al.*, 2007 ▶). Accordingly, the corresponding *X*—H bonds do not possess BCPs (Tables 2 ▶ and 3 ▶).

### 3.2. Atomic displacements and covalent bonding   

A multipole model is a better structure model than the IAM. This holds true for a multipole model with multipole parameters fixed to values from a database (Afonine *et al.*, 2004 ▶; Dittrich *et al.*, 2005 ▶, 2008 ▶). While atomic positions might differ by very small amounts between the multipole model and IAM, this generally means that ADPs in a multipole model are smaller than ADPs in the corresponding IAM. This feature is explained by electron density in covalent bonds being mimicked by increased values of ADPs, while it is explicitly present in the aspherical scattering factors of the multipole model. It is presently observed for HEWL, where the ADPs from the IAM are slightly larger than those from the ELMAM2 model, although the differences are small.

**Figure 3:**
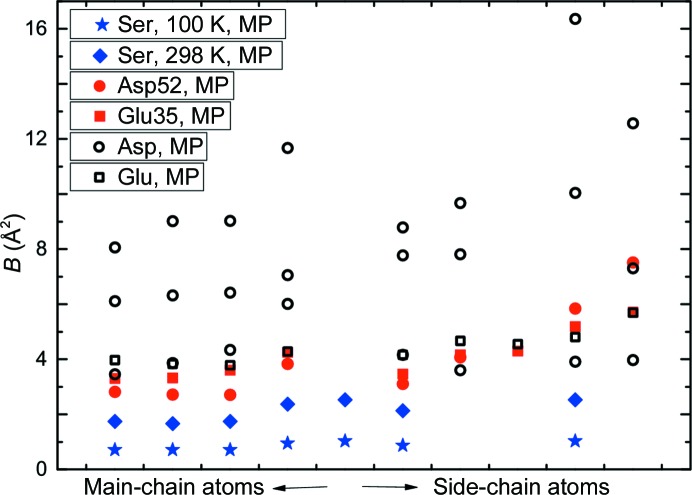
*B* factors of Glu35 (red filled squares), Asp52 (red filled circles), *B* factors of ordered glutamic acid (black open squares) and aspartic acid (black open circles) residues from HEWL and corresponding *B* factors for serine at 100 K (blue asterisks) and at 298 K (blue diamonds) (Mondal *et al.*, 2012 ▶). Values are from the particular multipole models.

The ADPs from the ELMAM2 model have magnitudes within the same range of values as possessed by the ADPs from the IAM published by Wang *et al.* (2007 ▶). While the sample temperature of HEWL was 100 K for the X-ray diffraction experiment, the ADPs are considerably larger than the ADPs of similar atoms in serine at 100 K and even larger than the ADPs in serine at 298 K (Mondal *et al.*, 2012 ▶; Fig. 3 ▶).

The discrepancy between expected amplitudes of atomic vibrations and values of ADPs can be explained by the intrinsic flexibility of proteins (Radivojac *et al.*, 2004 ▶; Yuan *et al.*, 2005 ▶; Weiss, 2007 ▶; He *et al.*, 2009 ▶). Although triclinic HEWL can be regarded as a rather rigid protein with a low solvent content and with low *B* factors compared with other HEWL crystal structures, approximately one third of the side chains exist in more than one conformation (Wang *et al.*, 2007 ▶). Such a feature can only be partially modelled by multiple positions of the atoms, and it will be one cause of the large values of the ADPs. Nevertheless, the large values of the ADPs apply to the ordered part of the ELMAM2 model of HEWL (Fig. 3 ▶). Since at 100 K water and other solvents will be frozen, these ADPs will reflect static disorder rather than atomic vibrations.

Whether the observed static disorder represents the flexibility of HEWL at room temperature or whether its reduced magnitude might be caused by the process of cooling cannot be determined from the X-ray diffraction data. However, ADP values that are larger than the values that are common in room-temperature structures of small-molecule crystals have the important implication that a meaningful refinement of multipole parameters cannot be performed (Dittrich *et al.*, 2009 ▶; Gatti & Macchi, 2012 ▶). This feature is reflected in the *R* values, which are only marginally better for the ELMAM2 model than for the IAM. This is not the result of the limited resolution of the diffraction data or possible poor quality of the intensity values (Kalinowski, 2010 ▶). As stated above, the large ADPs obliterate most if not all of the effects of chemical bonding on the electron density and thus on the diffraction. Hence, a simultaneous refinement of both ADPs and multipole parameters cannot be performed in a meaningful way.

Instead, the refinement of multipole models is only meaningful with the multipole parameters fixed to values from a database (Jelsch *et al.*, 1998 ▶; Dittrich *et al.*, 2008 ▶; Dominiak *et al.*, 2009 ▶; Domagała *et al.*, 2012 ▶), as performed here for the ELMAM2 model of HEWL. Apparently, this feature has been overlooked in previous electron-density studies on proteins, in which at least part of the multipole parameters have been refined (Jelsch *et al.*, 2000 ▶; Schmidt *et al.*, 2003 ▶, 2011 ▶; Guillot *et al.*, 2008 ▶).

Despite fixed database values for the multipole parameters, the static electron density of the ELMAM2 model does provide a good description of covalent bonds. As noticed, the BCPs of covalent non-hydrogen bonds are found at corresponding positions in ρ^stat^_MP_(**r**) and ρ^stat^_IAM_(**r**). Generally, ρ^stat^_MP_(BCP) > ρ^stat^_IAM_(BCP) (Tables 2 ▶ and 3 ▶). Laplacians at BCPs of covalent bonds are strongly negative for ρ^stat^_MP_(**r**), while values close to zero or even positive values are found for ∇^2^ρ^stat^_IAM_(BCP). Large values of ρ(BCP) and negative Laplacians of large magnitude are indicative of strong covalent bonding according to the QTAIM (Bader, 1994 ▶). The differences between the two static electron densities at their BCPs are the result of chemical bonding, which is solely contained in the ELMAM2 model. These features are similar to those found for small molecules (Mondal *et al.*, 2012 ▶; Prathapa *et al.*, 2013 ▶). They demonstrate the failure of the IAM for proper characterization of chemical bonding.

Primarily, the effect of thermal motion of the atoms on the electron density is to lower the local maxima, *i.e* ρ^dyn^(**r**) ≪ ρ^stat^(**r**) at the local maxima (Mondal *et al.*, 2012 ▶). Thermal averaging has only a moderate influence on the electron densities at BCPs, with ρ^dyn^_IAM_(BCP) ≃ ρ^stat^_IAM_(BDP) (Tables 2 ▶ and 3 ▶; see also Mondal *et al.*, 2012 ▶). The fact that ρ^dyn^_MP_(BCP) ≃ ρ^dyn^_IAM_(BCP) corroborates the observation that large ADPs lead to dynamic IAM and multipole densities in which most of the effects of chemical bonding are obliterated by the large distribution of the atomic positions (Fig. 2 ▶).

The Laplacians at the BCPs of dynamic electron densities strongly depend on the values of the ADPs (Mondal *et al.*, 2012 ▶). Here, we find positive values of large magnitude for ∇^2^ρ^dyn^_IAM_(BCP) ≃ ∇^2^ρ^dyn^_MP_(BCP), as opposed to small values or strongly negative values for ∇^2^ρ^stat^_IAM_(BCP) and ∇^2^ρ^stat^_MP_(BCP). These findings are on a par with those for small molecules (Mondal *et al.*, 2012 ▶).

### 3.3. Hydrogen bonds   

**Figure 4:**
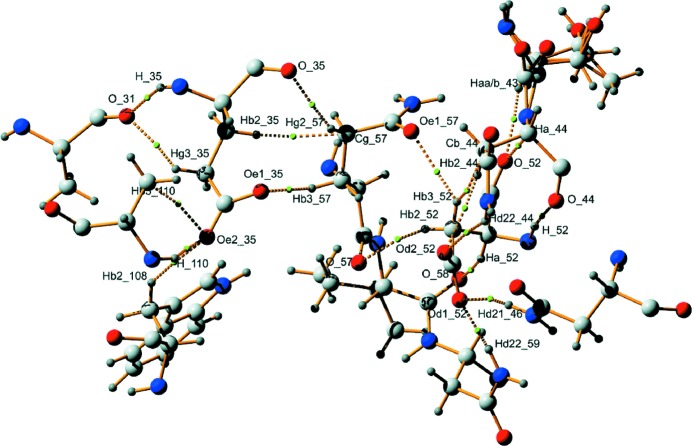
Schematic drawing of the hydrogen bonds formed by Glu35 and Asp52. Dashed lines represent hydrogen bonds and green spheres indicate their BCPs.

Fig. 4 ▶ provides a schematic drawing of the hydrogen bonds formed by Glu35 and Asp52, as presently identified through their BCPs in the static and dynamic electron densities.

Strynadka & James (1991 ▶) reported two hydrogen bonds for Glu35. Here, we confirm a hydrogen bond between the carboxyl group of Glu35 as an acceptor and the NH group of alanine Ala110 (O^∊2^_35⋯H_110—N_110). Instead of forming a hydrogen bond to a water molecule, the strongest hydrogen bond of Glu35 is between N_35—H_35 and O_31 of Ala31. The observed differences between hydrogen bonds in different structures of HEWL is probably owing to different solvent contents of the crystals used by Strynadka & James (1991 ▶) and Wang *et al.* (2007 ▶). In addition to these two hydrogen bonds, we find six weak hydrogen bonds of the type C—H⋯O (Table 2 ▶; Desiraju & Steiner, 2001 ▶). Although weak, they might be important for defining the stability of HEWL and the role of Glu35 in the catalytic process.

In agreement with Strynadka & James (1991 ▶), for Asp52 we find several hydrogen bonds involving O^δ1^_52 and O^δ2^_52, although solvent molecules are presently not involved (Table 3 ▶). Furthermore, we find an important hydrogen bond involving N_52—H_52 as a donor. Similar to Glu35, Asp52 is involved in six weak C—H⋯O hydrogen bonds, which might be important for the stability and role of Asp52 in the catalytic process.

A first indication about the strengths of hydrogen bonds is obtained from the values of the densities and Laplacians at the BCPs, whereby the static ELMAM2 density appears to be the most informative of the four electron densities considered here (Tables 2 ▶ and 3 ▶). The relations between properties at BCPs of the four electron densities are in agreement with those observed for small molecules (Mondal *et al.*, 2012 ▶). Values of up to ρ(BCP) = 0.24 e Å^−3^ indicate that all hydrogen bonds are weak or at best of intermediate strength. In agreement with results on small molecules, ∇^2^ρ(BCP) is small and positive, indicating a closed-shell character of the interaction (Bader, 1994 ▶).

**Figure 5:**
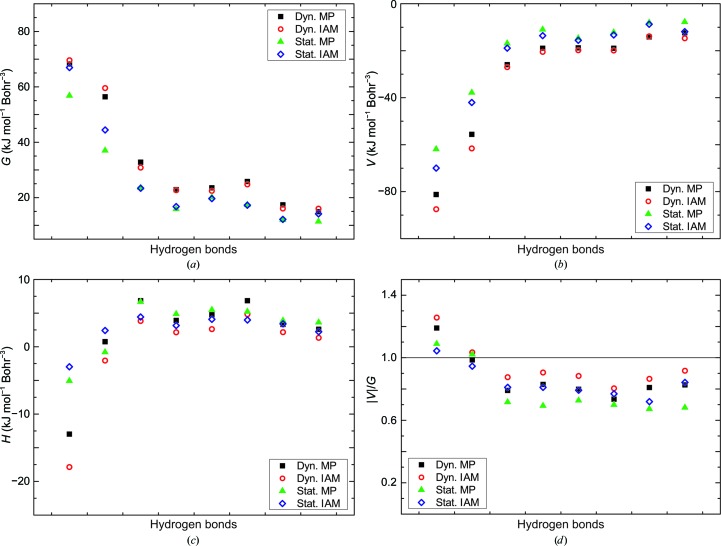
Energy densities at BCPs of hydrogen bonds of Glu35. (*a*) Kinetic energy density *G*(BCP), (*b*) potential energy density *V*(BCP), (*c*) total energy density *H*(BCP) = *G*(BCP) + *V*(BCP) and (*d*) the ratio |*V*(BCP)|/*G*(BCP). Black squares, values from ρ^dyn^_MP_(**r**); red circles, values from ρ^dyn^_IAM_(**r**); green triangles, values from ρ^stat^_MP_(**r**); blue diamonds, values from ρ^stat^_IAM_(**r**).

An estimate of the character of hydrogen bonds can be obtained from the energy densities at the BCPs (§2; Espinosa *et al.*, 1998 ▶). The ratio |*V*(BCP)|/*G*(BCP) < 1 indicates that most hydrogen bonds are weak and of mainly electrostatic nature (Espinosa *et al.*, 2002 ▶). Only O_31⋯N_35—H_35 involving Glu35 and O^δ1^_52⋯H^δ22^_59—N^δ2^_59 and O_44⋯H_52—N_52 involving Asp52 have |*V*(BCP)|/*G*(BCP) ≃ 1.1 (*i.e.* > 1) and total energy densities that are negative (Figs. 5 ▶ and 6 ▶). Accordingly, these three hydrogen bonds have a portion of covalency (Cremer & Kraka, 1984 ▶) or are of mixed covalent/ionic character, but they still are relatively weak hydrogen bonds compared with the many hydrogen bonds in small molecules.

### 3.4. Reaction mechanism   

The atomic charges of the atoms of Glu35 and Asp52 are of comparable magnitudes for ρ^dyn^_IAM_(**r**) and ρ^dyn^_MP_(**r**) (Table 4 ▶), although the latter should be considered to be more accurate. Integrated atomic charges could not be obtained by *VMoPro* for the static electron densities. As discussed in §3.1, H atoms are included in the atomic basins of the non-H atoms to which they are covalently bonded. Accordingly, charges are reported for NH, CH and CH_2_ groups for both the experimental and the theoretical static electron densities, as indicated in Table 4 ▶.

The atomic charges from ρ^dyn^_MP_(**r**) [and ρ^dyn^_IAM_(**r**) as well] follow chemical expectations, with positively charged C atoms and negatively charged O atoms and NH groups. These properties are essentially different from the theoretical electron densities of Godjayev *et al.* (1998 ▶), who proposed a negatively charged C^γ^ atom of the carboxyl group of Asp52 along with positively charged O atoms (Table 4 ▶). Although not impossible in principle, the present experimental electron densities lead to the scenario of O atoms of the carboxyl group that possess negative charges. This is of importance for the proposed catalytical mechanisms of HEWL. According to the Phillips mechanism (Phillips, 1966 ▶), negative charge of Asp52 is essential for the enzymatic reaction since it is supposed to stabilize the carbenium ion formed after the cleavage of the glycosidic bond.

However, the carboxyl group of Glu35 is not protonated in the present crystal structure, which may be owing to pH values during crystal growth that do not correspond to those of the active enzyme, or it may be the true state of Glu35 in HEWL.

Since this proton is an essential proton for the Phillips mechanism (Phillips, 1966 ▶), the chemical characters of Glu35 and Asp52 of the present structure of HEWL are consistent with both the Phillips mechanism (Phillips, 1966 ▶) and the Koshland mechanism (Koshland, 1953 ▶), prohibiting a clear approval of one or the other reaction mechanism.

## 4. Conclusions   

Wang *et al.* (2007 ▶) have noticed that HEWL is rather rigid for a crystallized protein molecule, but that it nevertheless contains disordered parts for about 35% of the structure. Here, we have found that the ADPs of atoms within ordered parts of the structure have magnitudes that are larger than the magnitudes of the ADPs of small molecules in their crystallized state at room temperature, despite the nominal temperature of 100 K of HEWL (Fig. 3 ▶). Therefore, the ADPs of HEWL at 100 K reflect frozen static disorder rather than thermal vibrations (§[Sec sec3.2]3.2).

It its generally accepted that multipole refinements provide meaningful values for multipole parameters only if the magnitudes of the ADPs reflect thermal vibrations of the atoms at temperatures of 100 K and below (Gatti & Macchi, 2012 ▶). An important consequence of the observed large ADPs is thus to prevent meaningful refinement of the multipole model, despite reports of such refinements for some proteins in the literature (Jelsch *et al.*, 2000 ▶; Schmidt *et al.*, 2003 ▶, 2011 ▶; Guillot *et al.*, 2008 ▶).

Instead, useful information about the redistribution of electron density owing to chemical bonding can be obtained from refinements of atomic coordinates and ADPs of structure models incorporating multipole parameters fixed to values from a database of transferable multipole parameters, as is presented here for the ELMAM2 model of HEWL (Jelsch *et al.*, 1998 ▶; Dittrich *et al.*, 2008 ▶; Dominiak *et al.*, 2009 ▶; Domagała *et al.*, 2012 ▶).

We present and analyse static and dynamic electron densities based on a multipole model of HEWL. The large ADPs are reflected in the dynamic electron densities, such that the dynamic IAM and ELMAM2 densities are similar within their low-density regions, despite clear differences between the corresponding static densities (Tables 2 ▶ and 3 ▶). The flexibility of a protein is visible as distinct differences between static and dynamic densities. However, the quantitative interpretation of topological properties of dynamic densities is beyond the present state of the art of charge-density analysis (Mondal *et al.*, 2012 ▶).

Consideration of the static ELMAM2 density has shown that electron densities in covalent bonds have similar properties to those in small biological molecules. The analysis of intramolecular interactions involving the Glu35 and Asp52 residues has revealed the presence of N—H⋯O hydrogen bonds of intermediate strength, in agreement with the literature. The presence of BCPs in the ELMAM2 density as indicator for chemical interactions between atoms has revealed many weak C—H⋯O hydrogen bonds, which might be important for the stability and function of this protein.

The topological analysis of the ELMAM2 density has lead to negative charges on the carboxylic O atoms of Asp52, thus supporting the Phillips mechanism for the catalytic activity of HEWL (Phillips, 1966 ▶). However, the deprotonated state of the carboxyl group of Glu35 makes the present electron densities in agreement with both the Koshland and Phillips mechanisms (Koshland, 1953 ▶; Phillips, 1966 ▶), and they cannot be distinguished here. Electron densities of one or more complexes of HEWL with a substrate or inhibitor and an electron density of HEWL with Glu35 in the protonated state might allow a unique assignment of the mechanism and might lead to a more detailed characterization of the catalytic mechanism.

## Figures and Tables

**Figure 6:**
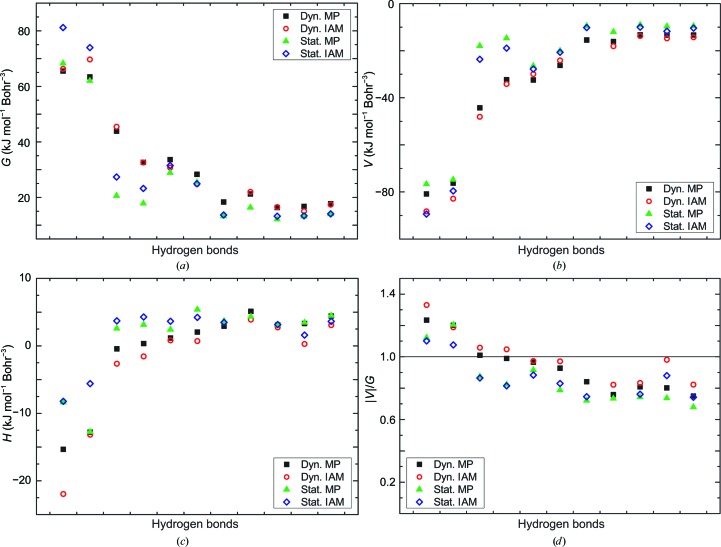
Energy densities at BCPs of hydrogen bonds of Asp52. (*a*) Kinetic energy density *G*(BCP), (*b*) potential energy density *V*(BCP), (*c*) total energy density *H*(BCP) = *G*(BCP) + *V*(BCP) and (*d*) the ratio |*V*(BCP)|/*G*(BCP). Black squares, values from ρ^dyn^_MP_(**r**); red circles, values from ρ^dyn^_IAM_(**r**); green triangles, values from ρ^stat^_MP_(**r**); blue diamonds, values from ρ^stat^_IAM_(**r**).

**Table 1:** Crystallographic data of hen egg-white lysozyme (PDB entry 2vb1; Wang *et al.*, 2007 ▶)

Space group	*P*1
*Z*	1
*a* (Å)	27.07
*b* (Å)	31.25
*c* (Å)	33.76
α (°)	87.98
β (°)	108.00
γ (°)	112.11
*V* (Å^3^)	25057.0
*F*(000)	10268.0
*T* (K)	100
Wavelength (Å)	0.65
[sin(θ)/λ]_max_ (Å^−1^)	0.77
*d*_min_ (Å)	0.65
pH	4.7
Multiplicity	7.1
*R*_merge_ (%)	4.00
Completeness (%)	97.6
Unique reflections (observed/all)	166259/187165
*R*_all_ (%)	8.39
IAM before elongation of bond lengths†	
*R*_F_ [*F*_obs_ with |*I*_obs_ − *I*_calc_|/σ(*I*_obs_) < 45/all reflections] (%)	8.39/8.54
*R*_wF_ [*F*_obs_ with |*I*_obs_ − *I*_calc_|/σ(*I*_obs_) < 45/all reflections] (%)	10.19/10.80
Final IAM†	
*R*_F_ [*F*_obs_ with |*I*_obs_ − *I*_calc_|/σ(*I*_obs_) < 45/all reflections] (%)	8.39/8.54
*R*_wF_ [*F*_obs_ with |*I*_obs_ − *I*_calc_|/σ(*I*_obs_) < 45/all reflections] (%)	10.19/10.80
ELMAM2 model†	
*R*_F_ [*F*_obs_ with |*I*_obs_ − *I*_calc_|/σ(*I*_obs_) < 45] (%)	8.07
*R*_wF_ [*F*_obs_ with |*I*_obs_ − *I*_calc_|/σ(*I*_obs_) < 45] (%)	9.88

† Agreement indices from the IAM and the ELMAM2 model are from the present work.

**Table 2:** Topological properties at bond critical points (BCPs) of Glu35 from static and dynamic densities. First line, ρ(BCP) (e Å^−3^); second line, ∇^2^ρ(BCP) (e Å^−5^)

Bond	ρ^stat^_IAM_(**r**)	ρ^stat^_MP_(**r**)	ρ^dyn^_IAM_(**r**)	ρ^dyn^_MP_(**r**)
C^*n*−1^—N	1.81	2.29	1.75	1.76
	−4.00	−23.48	17.38	15.87
N—H	1.84	2.30	—	—
	−17.35	−39.03	—	—
C^α^—N	1.48	1.75	1.58	1.59
	2.14	−9.81	12.33	12.21
C^α^—H^α^	1.47	1.87	—	—
	−8.70	−18.66	—	—
C^α^—C	1.19	1.61	1.38	1.46
	1.31	−9.12	10.65	8.35
C—O	2.14	2.71	2.53	2.30
	6.91	−23.98	38.26	41.75
C—N^*n*+1^	1.76	2.23	1.75	1.74
	−2.94	−21.19	20.16	17.77
C^α^—C^β^	1.19	1.59	1.18	1.12
	1.55	−8.78	4.18	5.03
C^β^—H^β2^	1.47	1.81	—	—
	−8.74	−16.94	—	—
C^β^—H^β3^	1.47	1.80	—	—
	−8.74	−16.95	—	—
C^β^—C^γ^	1.24	1.61	1.42	1.41
	1.09	−9.33	11.13	10.98
C^γ^—H^γ2^	1.47	1.81	—	—
	−8.75	−16.93	—	—
C^γ^—H^γ3^	1.47	1.81	—	—
	−8.74	−16.93	—	—
C^γ^—C^δ^	1.21	1.70	1.30	1.28
	1.20	−11.45	14.15	14.91
C^δ^—O^∊1^	1.92	2.58	3.12	3.07
	−3.11	−30.88	13.93	16.90
C^δ^—O^∊2^	2.15	2.82	2.92	2.89
	8.12	−24.18	28.54	32.00
O_31⋯H_35—N_35	0.22	0.21	0.27	0.25
	2.35	1.90	1.90	2.03
O^∊2^_35⋯H_110—N_110	0.15	0.15	0.20	0.18
	1.72	1.33	2.11	2.10
O_31⋯H^γ3^_35—C^γ^_35	0.08	0.07	0.11	0.10
	1.02	1.11	1.27	1.45
O^∊2^_35⋯H^β3^_110—C^β^_110	0.07	0.05	0.09	0.08
	0.73	0.76	0.91	0.98
O_35⋯H^γ2^_57—C^γ^_57	0.07	0.06	0.09	0.08
	0.87	0.94	0.92	1.04
O^∊1^_35⋯H^β3^_57—C^β^_57	0.06	0.05	0.08	0.07
	0.78	0.83	1.09	1.20
O^∊2^_35⋯H^β2^_108—C^β^_108	0.04	0.04	0.07	0.07
	0.57	0.58	0.67	0.76
C^γ^_57⋯H^β2^_35—C^β^_35	0.06	0.04	0.08	0.06
	0.60	0.55	0.64	0.64

**Table 3:** Topological properties at bond critical points (BCPs) of Asp52 from static and dynamic densities. First line, ρ(BCP) (e Å^−3^); second line, ∇^2^ρ(BCP) (e Å^−5^)

Bond	ρ^stat^_IAM_(**r**)	ρ^stat^_MP_(**r**)	ρ^dyn^_IAM_(**r**)	ρ^dyn^_MP_(**r**)
C^*n*−1^—N	1.79	2.27	1.77	1.80
	−3.56	−22.55	10.37	7.81
N—H	1.84	2.30	—	—
	−17.36	−39.04	—	—
C^α^—N	1.50	1.78	1.50	1.52
	1.81	−10.53	8.33	8.51
C^α^—H^α^	1.46	1.87	—	—
	−8.70	−18.77	—	—
C^α^—C	1.18	1.59	1.21	1.31
	1.51	−8.74	4.88	1.98
C—O	2.13	2.70	2.39	2.13
	6.14	−24.80	26.69	30.77
C—N^*n*+1^	1.80	2.28	1.73	1.76
	−3.68	−22.84	12.79	10.30
C^α^—C^β^	1.20	1.60	1.22	1.15
	1.46	−8.98	4.64	5.76
C^β^—H^β2^	1.47	1.81	—	—
	−8.75	−16.93	—	—
C^β^—H^β3^	1.47	1.81	—	—
	−8.74	−16.94	—	—
C^β^—C^γ^	1.22	1.72	1.20	1.18
	1.17	−11.91	7.12	8.56
C^γ^—O^δ1^	2.20	2.84	2.63	2.49
	11.55	−22.76	27.19	30.21
C^γ^—O^δ2^	2.06	2.72	2.66	2.53
	1.64	−32.92	35.40	37.50
O^δ1^_52⋯H^δ22^_59—N^δ2^_59	0.26	0.24	0.28	0.25
	2.68	2.20	1.63	1.84
O_44⋯H_52—N_52	0.24	0.24	0.25	0.24
	2.51	1.81	2.08	1.86
O^δ1^_52⋯H^δ21^_46—N^δ2^_46	0.10	0.08	0.17	0.16
	1.14	0.85	1.57	1.60
O^δ2^_52⋯H^δ22^_44—N^δ2^_44	0.08	0.07	0.14	0.13
	1.01	0.77	1.14	1.21
O_52⋯Ha_44—N_44	0.11	0.11	0.12	0.13
	1.29	1.15	1.16	1.28
O_52⋯H^α^a/b_43—C^α^_43	0.09	0.08	0.11	0.11
	1.07	1.13	0.94	1.11
O^δ2^_52⋯H^β2^_44—C^β^_44	0.05	0.05	—	0.08
	0.63	0.62	—	0.78
O_58⋯H^α^_52—C^α^_52	—	0.06	0.08	0.07
	—	0.76	0.95	0.97
O_57⋯H^β2^_52—C^β^_52	0.05	0.05	0.07	0.07
	0.60	0.56	0.71	0.71
C^β^_44⋯H^β3^_52—C^β^_52	0.07	0.05	0.08	0.07
	0.55	0.61	0.56	0.74
O^∊1^_57⋯H^β3^_52—C^β^_52	0.05	0.04	0.07	0.06
	0.65	0.68	0.75	0.82

**Table 4:** Net atomic charges *Q* of Glu35 and Asp52 from dynamic densities and from quantum-chemical calculations (Godjayev *et al.*, 1998 ▶). For dynamic densities H atoms are included within the atomic basin of their neighbouring atom owing to the absence of a local atomic maxima for H atoms

Atom	*Q* ρ^dyn^_IAM_(**r**)	*Q* ρ^dyn^_MP_(**r**)	*Q* quant.
Glu35			
N(H)	−0.33	−0.33	−0.36
C^α^(H^α^)	0.29	0.18	−0.06
C	0.40	0.37	0.45
O	−0.38	−0.52	−0.38
C^β^(H^β2^, H^β3^)	0.01	0.07	−0.03
C^γ^(H^γ2^, H^γ3^)	0.09	0.12	−0.11
C^δ^	0.48	0.33	0.50
O^∊1^	−0.32	−0.58	−0.36
O^∊2^	−0.34	−0.66	−0.35
H	—	—	0.18
H^α^	—	—	0.02
H^β2^	—	—	0.03
H^β3^	—	—	0.03
H^γ2^	—	—	0.06
H^γ3^	—	—	0.06
H^∊2^	—	—	0.21
Asp52			
N(H)	−0.40	−0.42	−0.36
C^α^(H^α^)	0.24	0.22	−0.06
C	0.55	0.48	0.45
O	−0.47	−0.61	−0.30
C^β^(H^β2^, H^β3^)	0.08	0.05	−0.17
C^γ^	0.61	0.43	−0.50
O^δ1^	−0.42	−0.62	0.57
O^δ2^	−0.37	−0.66	0.57
H	—	—	0.18
H^α^	—	—	0.02
H^β2^	—	—	0.02
H^β3^	—	—	0.02
